# Supplementary figures and images for: Assessment of the Histone Mark-based Epigenomic Landscape in Human Myometrium at Term Pregnancy
Source: bioRxiv. 2025 Feb 26:2024.02.19.581035. Preprint. [Version 2] doi: 10.1101/2024.02.19.581035 (PMC11888205; doi:10.1101/2024.02.19.581035)

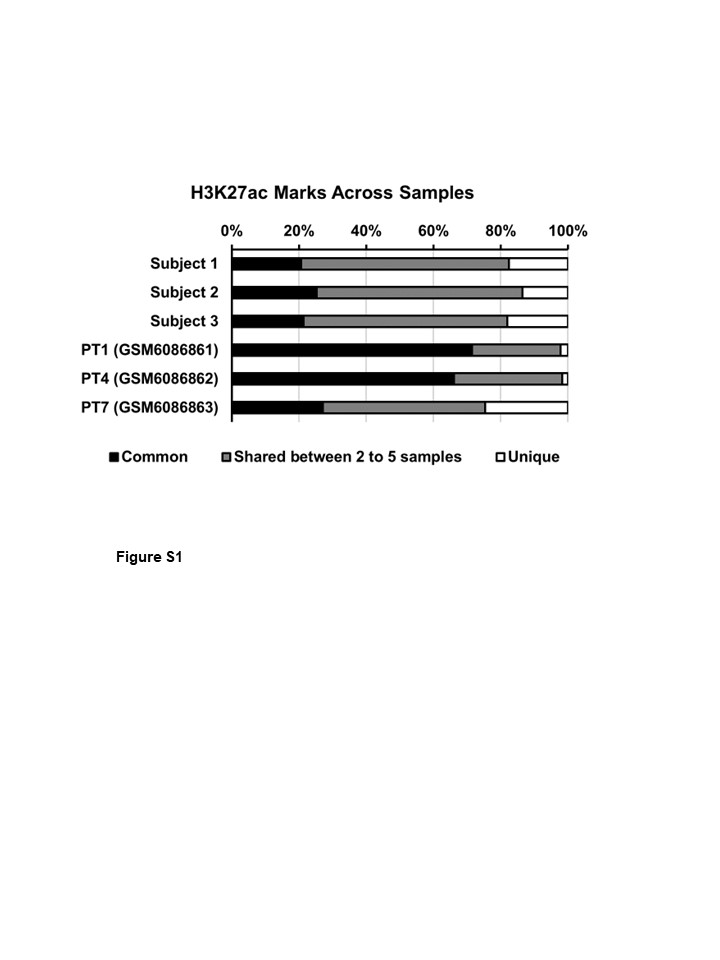

Supplement: Supplement 10 — Figure S1. Subject variations on H3K27ac-positive histone marks. [file media-10.jpg]

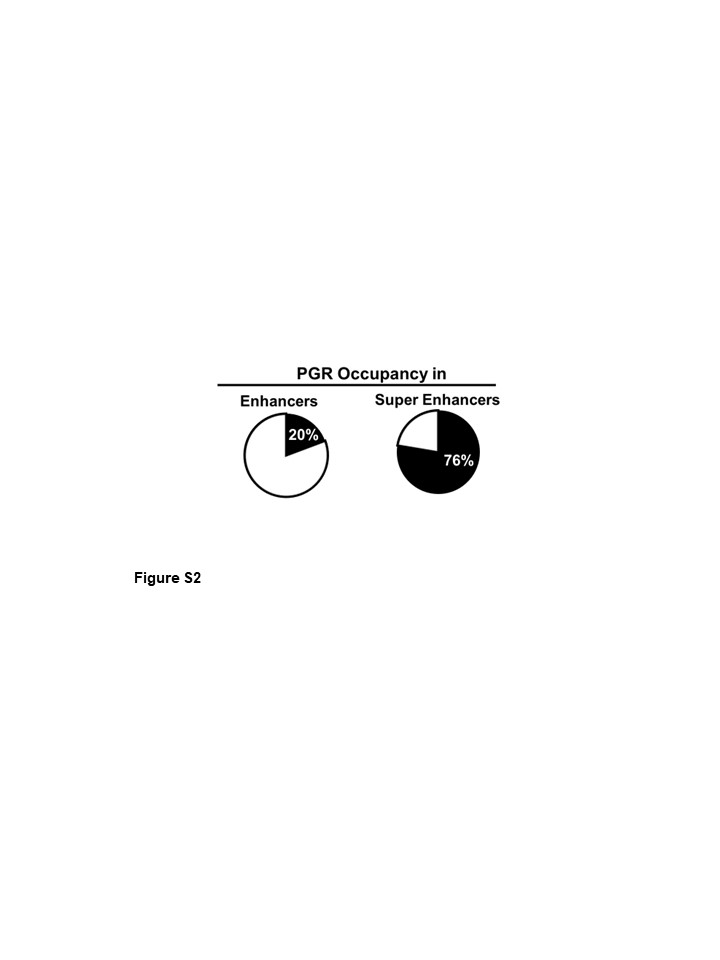

Supplement: Supplement 11 — Figure S2. PGR occupancy in myometrial enhancers. (A) Percentages of enhancers and super enhancers that manifest PGR occupancy. PGR genome occupancy data was previously published in NCBI GEO accession numbers GSM4081683 and GSM4081684. [file media-11.jpg]
